# Evaluation of Social Isolation Trajectories and Incident Cardiovascular Disease Among Middle-Aged and Older Adults in China: National Cohort Study

**DOI:** 10.2196/45677

**Published:** 2023-06-30

**Authors:** Lan Guo, Wanxin Wang, Jingman Shi, Xinyu Zheng, Yilin Hua, Ciyong Lu

**Affiliations:** 1 School of Public Health Sun Yat-sen University Guangzhou China

**Keywords:** social isolation trajectories, cardiovascular disease, CVD, cohort study, middle-aged and older adults, China, social isolation

## Abstract

**Background:**

Although the association between social isolation and the risk of subsequent cardiovascular disease (CVD) is well documented, most studies have only assessed social isolation at a single time point, and few studies have considered the association using repeatedly measured social isolation.

**Objective:**

This study aimed to examine the association between social isolation trajectories and incident CVD in a large cohort of middle-aged and older adults.

**Methods:**

This study used data from 4 waves (wave 1, wave 2, wave 3, and wave 4) of the China Health and Retirement Longitudinal Study. We defined the exposure period as from June 2011 to September 2015 (from wave 1 to wave 3) and the follow-up period as from September 2015 to March 2019 (wave 4). On the basis of the inclusion and exclusion criteria, our final analytic sample included 8422 individuals who had no CVD in the China Health and Retirement Longitudinal Study in waves 1 to 3 and were fully followed up in wave 4. Social isolation was ascertained using an extensively used questionnaire at 3 consecutive, biennial time points from waves 1 to 3, and individuals were assigned to 3 predefined social isolation trajectories based on their scores at each wave (consistently low, fluctuating, and consistently high). Incident CVD included self-reported physician-diagnosed heart disease and stroke combined. Cox proportional hazard models estimated the associations of social isolation trajectories with risks of incident CVD, adjusting for demographics, health behaviors, and health conditions.

**Results:**

Of the 8422 participants (mean age 59.76, SD 10.33 years at baseline), 4219 (50.09%) were male. Most of the participants (5267/8422, 62.54%) had consistently low social isolation over time and 16.62% (1400/8422) of the participants had consistently high social isolation over the exposure period. During the 4-year follow-up, 746 incident CVDs occurred (heart disease: 450 cases and stroke: 336 cases). Compared with individuals with consistently low social isolation, those with fluctuating social isolation (adjusted hazard ratio 1.27, 95% CI 1.01-1.59) and consistently high social isolation (adjusted hazard ratio 1.45, 95% CI 1.13-1.85) had higher risks for incident CVD after adjusting for demographics (ie, age, sex, residence, and educational level), health behaviors (ie, smoking status and drinking status), and health conditions (ie, BMI; history of diabetes, hypertension, dyslipidemia, chronic kidney disease; use of diabetes medications, hypertension medications, and lipid-lowering therapy; and depressive symptoms scores).

**Conclusions:**

In this cohort study, middle-aged and older adults with fluctuating and consistently high social isolation exposure had higher risks of the onset of CVD than those without the exposure. The findings suggest that routine social isolation screenings and efforts to improve social connectedness merit increased attention for preventing CVD among middle-aged and older adults.

## Introduction

### Background

Social isolation, defined as an objective reduction in social network size and frequency of social contact, has been shown to be a critical factor contributing to physical and mental health risks [1]. Previous evidence has suggested that it is particularly common in older adults, who may experience decreasing economic resources, physical impairment, or the loss of contemporaries, all of which can limit social contact [2,3]. As the global population ages, there has been an increasing interest in assessing the health effects of social isolation in older adults. The impact of COVID-19 has amplified concerns regarding social isolation among older adults, leading to greater public health awareness [4,5]. In China, it is projected that the proportion of empty-nest families (referring to older individuals with no children or whose children have already left home) will reach 90% [6], highlighting the need for increased attention to social isolation among middle-aged and older adults.

Cardiovascular disease (CVD) is one of the leading causes of global mortality and a major contributor to disability in older adults [7]. Several epidemiological studies have examined the association between social isolation and CVD; however, the results have been inconsistent. Some prospective studies have found that social isolation was associated with a higher risk of developing CVDs, such as coronary heart disease [8], heart failure [9], and stroke [10]. A meta-analysis with 16 cohorts from Europe, North America, Australia, Japan, and Asian Russia showed that social isolation and loneliness are associated with approximately 30% higher risk of coronary heart disease and stroke, even after controlling for age, sex, and socioeconomic status [3]. However, a study that included individuals from the UK Biobank found that the association between social isolation and incident acute myocardial infarction or stroke was attenuated after considering other risk factors, including biological factors (BMI, diastolic and systolic blood pressure, and grip strength), health behaviors (alcohol consumption, physical activity, and smoking), depressive symptoms, socioeconomic factors (education, household income, and Townsend Deprivation Index), and history of chronic illness [11]. A study using data from the English Longitudinal Study of Ageing (ELSA) did not observe a significant association between social isolation and incident CVD over a mean follow-up period of 5.4 years [12]. Similarly, another meta-analysis, including 5 longitudinal studies from Australian adults, reported no association between social isolation and the incidence of CVD [13]. Moreover, there is increasing evidence that social isolation is associated with CVD risk factors such as increased hypertension, smoking, psychological factors (eg, depression), and biological mechanisms (eg, inflammation and stress reactivity) [14-16]. It has been well established that lifestyle [17] and health conditions [18] are associated with an increased risk of CVD outcomes, and therefore, the confounding or covariate influences of these factors on the association between social isolation and incident CVD should be considered when estimating the aforementioned association between social isolation and CVD.

Moreover, the level of exposure to social isolation may be dynamic and change over time, with some individuals experiencing long-term social isolation, whereas others experiencing a temporary increase in social isolation [19]. However, prior studies evaluating the association between social isolation and CVD have used social isolation at only a single time point [8-11], which may not provide a complete picture of the role of social isolation in CVD risk. An in-depth understanding of social isolation trajectories may provide clues for identifying individuals with different time-varying levels (eg, consistently high, fluctuating, or consistently low) over their life course. To reduce the risk of CVD in middle-aged and older people, it is crucial to identify its association with social isolation to facilitate research into the development of targeted prevention or early intervention programs.

### Objectives

Therefore, to address this research gap, using the nationally representative data from the China Health and Retirement Longitudinal Study (CHARLS), we conducted longitudinal analyses to assess the associations of social isolation trajectories over a period of 4 years with incident CVD among the middle-aged and older Chinese population.

## Methods

### Study Population

**Figure 1:**
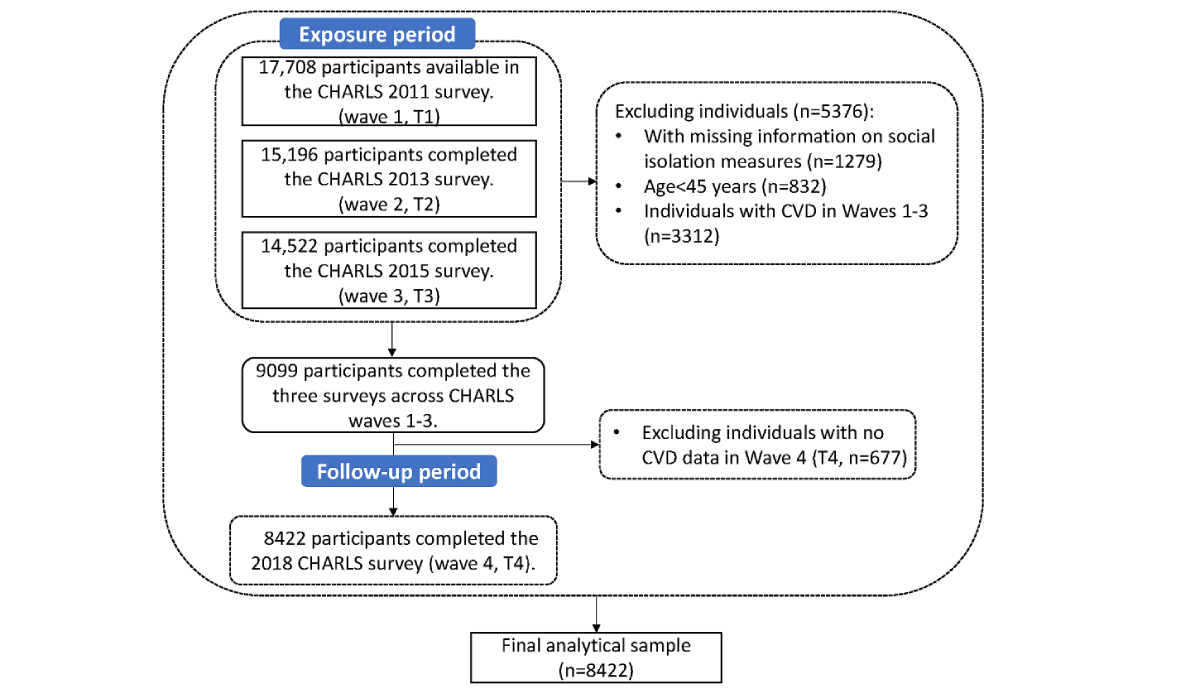
Flow diagram of participants included in the study. A total of 14,522 individuals had data available for waves 1-3. Individuals could be excluded due to multiple reasons. CHARLS: China Health and Retirement Longitudinal Study; CVD: cardiovascular disease.

This cohort study used data from CHARLS, an ongoing nationally representative cohort study of Chinese adults aged ≥45 years and their families, which has been previously described in detail [20]. The baseline survey was conducted between June 2011 and March 2012 using a multistage stratified probability-proportional-to-size (PPS) sampling method. In the first stage, 150 county-level units were randomly selected using a PPS sampling technique from a sampling frame containing all county-level units except Tibet. The sample was stratified by region and within each region by urban districts or rural counties and per capita gross domestic product statistics. The final sample of 150 counties was distributed across 28 provinces. The study sample used the lowest level of government organization consisting of administrative villages (*cun* in Chinese) in rural areas and neighborhoods (*shequ* or *juweihui* in Chinese) in urban areas as primary sampling units. A total of 3 primary sampling units within each county-level unit were selected using PPS sampling. At baseline, face-to-face computer-assisted personal interview data were collected from 17,708 participants in 10,257 households, who were recruited from 450 villages and resident communities across China. The response rate of the baseline survey was 80.5%. All participants were followed up every 2 years after the baseline survey using a face-to-face computer-assisted personal interview [20]. The CHARLS data include 4 waves of data (June 2011 to March 2019). We defined from June 2011 to September 2015 (from T1 to T3) as our exposure period (where T1 refers to wave 1, T2 to wave 2, and T3 to wave 3) and from September 2015 to March 2019 (T4, wave 4) as our follow-up period. On the basis of our inclusion and exclusion criteria, our final analytic sample included 8422 individuals who did not have CVD in CHARLS in waves 1 to 3 and were fully followed up in wave 4. Detailed process of participant selection is presented in Figure 1.

### Primary Exposure—Social Isolation Trajectories

In each biennial questionnaire, a social isolation index was generated based on social network ties and social activity or engagement [21]. Consistent with previous literature [22,23], the index consists of 4 indicators: currently unmarried (never married, separated, divorced, and widowed), living alone, having less than weekly contact (face-to-face, telephone, or email) with their children, and participating in social activities less than once a month. Detailed definitions of each indicator are provided in Methods in Multimedia Appendix 1 [22-27]. By summing the indicators, the total score ranged from 0 to 4, with higher scores indicating a greater level of social isolation. We performed Bartley sphericity test to analyze the validity structure of the social isolation index, and the *P* values across the first 3 waves were <.001. The rejection of the null hypothesis of Bartley sphericity test suggests that the validity structure of the social isolation index was good [28]. Participants were further categorized as nonisolated (score <2) and isolated (score ≥2) [22,23,29]. We created social isolation trajectories using the dichotomized social isolation variable for each wave (isolated vs nonisolated) [30]. Guided by theory and prior empirical work, we constructed 3 social isolation trajectories (consistently low, fluctuating, and consistently high) a priori based on combinations of social isolation status across the first 3 waves (wave 1 to 3) [31]. Consistently high was defined as isolated at 3 time points across the assessment period, consistently low was defined as nonisolated at 3 time points, and fluctuating encompassed the other social isolation trajectories that did not fit the above classifications. A visual depiction of the trajectories is provided in Table S1 in Multimedia Appendix 1.

### CVD Outcomes

Incident CVD events were defined as the first occurrence of CVD during the follow-up period. In line with previous literature using CHARLS data [32,33], incident CVD events, including heart disease and stroke, were measured using the following standardized questions: “Have you been told by a doctor that you have been diagnosed with a heart attack, coronary heart disease, angina, congestive heart failure, or other heart problems?” or “Have you been told by a doctor that you have been diagnosed with a stroke?” Participants who reported heart disease or stroke during the follow-up period were defined as having an incident CVD.

### Covariates

At baseline, information on the sociodemographic status and health-related factors was collected by trained interviews using a structured questionnaire. Demographic variables included age, sex, area of residence (rural or urban), and educational level (no formal education, primary school or below, middle or high school, and college or above). Health-related factors included smoking and drinking status (never, former, and current), BMI, depressive symptoms, hypertension, diabetes, dyslipidemia, chronic kidney disease, and the use of medications or therapies for hypertension, diabetes, and dyslipidemia. Detailed definitions of the covariates are provided in Multimedia Appendix 1.

Depressive symptoms were assessed using the 10-item Center for Epidemiology Scale for Depression (CESD-10), which has been validated and widely used among Chinese adults [34]. The CESD-10 consists of 10 items: (1) bothered by little things, (2) had trouble concentrating, (3) felt depressed, (4) everything was an effort, (5) felt hopeful, (6) felt fearful, (7) sleep was restless, (8) felt happy, (9) felt lonely, and (10) could not get going. Each depressive symptom item in the past week was measured from 0 (rarely or none of the time [<1 day]) to 3 (most or all of the time [5-7 days]). The sum of CESD-10 scores ranges from 0 to 30, with higher scores indicating a higher level of depressive symptoms severity. A total score of ≥12 was used as the cutoff for having depressive symptoms [35]. Of note, subjective loneliness was measured using the CESD-10.

A subgroup of 5196 CHARLS participants underwent measurements of metabolic biomarkers, including fasting plasma glucose, total cholesterol, low-density lipoprotein cholesterol, high-density lipoprotein cholesterol, triglycerides, high-sensitivity C-reactive protein (hs-CRP), and serum creatinine. The estimated glomerular filtration rate was calculated using the Chronic Kidney Disease Epidemiology Collaboration’s 2009 creatinine equation [36].

### Statistical Analysis

Data were described as mean (SD) or median (IQR) for continuous variables, and frequency with percentage was used to describe categorical variables. First, baseline characteristics were summarized based on the social isolation trajectory group and compared between participants using the chi-square test, ANOVA, or Kruskal-Wallis test, as appropriate. Second, we computed the incidence rates of CVD per 1000 person-years in CHARLS. We also calculated the follow-up time as the time elapsed from the date of the last interview, either the date of CVD diagnosis or the date of the latest interview (March 2019) in which the individual participated. Cox proportional hazard models estimated the hazard ratios (HRs) and 95% CIs for the associations between the social isolation trajectory group and incident CVD using the consistently low social isolation trajectory as the reference. Four models were estimated: model 1, an unadjusted model; in model 2, age and sex were adjusted; in model 3, age, sex, residence, educational level, smoking status, and drinking status were adjusted; and in model 4, the variables in model 3 plus BMI; history of diabetes, hypertension, dyslipidemia, and chronic kidney disease; use of diabetes medications, hypertension medications, and lipid-lowering therapy; and depressive symptoms scores were adjusted. We used interaction items and subgroup analyses to assess whether the potential association between the social isolation trajectory group and incident CVD was moderated by the following characteristics: age, sex, residence, educational level, smoking status, drinking status, diabetes, hypertension, dyslipidemia, chronic kidney disease, BMI, and depressive symptom scores. Four sensitivity analyses were conducted to assess the robustness of our study findings: (1) we further adjusted for metabolic biomarkers in the subgroup of 5196 participants who underwent metabolic examinations, (2) we additionally adjusted for health conditions and behaviors at wave 3 (T3) to estimate changes in associations due to potentially mediating health behavioral pathways, (3) we repeated analyses using the complete data set (7472 participants), and (4) we used inverse-probability weighting to examine the impact of potential selection bias by excluding individuals based on our exposure period [37]. All analyses were conducted using Stata (version 17.0; StataCorp) and R (version 4.2.1; R Foundation for Statistical Computing). Two-sided *P*<.05 was considered statistically significant.

### Ethics Approval

The CHARLS study was approved by the Biomedical Ethics Review Committee of Peking University (IRB00001052-11015), and ethics approval for the use of the CHARLS data was obtained from the University of Newcastle Human Research Ethics Committee. This study was conducted in accordance with the Strengthening the Reporting of Observational Studies in Epidemiology reporting guidelines. All procedures were performed in accordance with the principles of the Declaration of Helsinki. Written informed consent was obtained from all the participants.

## Results

### Characteristics of Participants

Of the 8422 participants included in the primary analyses, the mean age of the study population at baseline was 59.76 (SD 10.33) years, and the proportion of male participants was 50.09% (4219/8422). Although the differences between included and excluded participants were relatively small, participants excluded from analyses were older and were more likely to be female; live in rural areas; and have a lower educational level, higher depressive symptoms scores, and lower BMI (Table S2 in Multimedia Appendix 1). Table 1 describes the characteristics of the participants according to their social isolation trajectory group. Among these middle-aged and older adults, the majority (5267/8422, 62.54%) had consistently low social isolation over time and 16.62% (1400/8422) had consistently high social isolation over the exposure period. Individuals with consistently high versus consistently low social isolation were more likely to be older (mean age 66.08, SD 10.55 years vs mean age 58.41, SD 9.88 years; *P*<.001), be female (811/1400, 57.93% vs 2592/5267, 49.21%; *P*<.001), live in rural area (1241/1400, 88.64% vs 3868/5267, 73.44%; *P*<.001), have no formal education (908/1400, 64.86% vs 2029/5267, 38.52%; *P*<.001), be a nonsmoker (855/1400, 61.07% vs 3130/5267, 59.42%; *P*=.04), be a nondrinker (897/1400, 64.07% vs 3148/5267, 59.77%; *P*=.002), have higher CESD-10 scores (mean 10.41, SD 6.70 vs mean 7.67, SD 5.98), have a history of hypertension (602/1400, 43% vs 1866/5267, 35.43%; *P*<.001), less use of diabetes mediations (29/1400, 2.07% vs 184/5267, 3.49%; *P*=.02), have higher systolic pressure (mean 135.65, SD 27.09 mm Hg vs mean 130.36, SD 24.88 mm Hg), have lower triglyceride (mean 118.71, SD 96.77 mg/dL vs mean 131.32, SD 101.87 mg/dL; *P*=.002) but higher high-density lipoprotein cholesterol (mean 54.11, SD 15.94 mg/dL vs mean 50.93, SD 15.28 mg/dL; *P*<.001), and have a lower estimated glomerular filtration rate (mean 88.42, SD 28.99 mL/min/1.73 m^2^ vs mean 91.98, SD 36.09 mL/min/1.73 m^2^; *P*=.02).

**Table 1:** Characteristics of the participants according to social isolation trajectory group.

Characteristics			Overall (N=8422)		Social isolation trajectory group							*P* value^a^
					Consistently low (n=5267)		Fluctuating (n=1755)		Consistently high (n=1400)			
Age (years), mean (SD)			59.76 (10.33)		58.41 (9.88)		58.78 (9.57)		66.08 (10.55)		<.001	
Sex, male, n (%)			4219 (50.09)		2675 (50.79)		955 (54.41)		589 (42.07)		<.001	
**Area of residence^b^, n (%)**												<.001
	Rural	6617 (78.57)		3868 (73.43)		1508 (85.93)		1241 (88.64)				
	Urban	1803 (21.41)		1398 (26.54)		247 (14.07)		158 (11.28)				
**Educational level^b^, n (%)**												<.001
	No formal education	3698 (43.91)		2029 (38.52)		761 (43.36)		908 (64.86)				
	Primary school or below	1936 (22.99)		1213 (23.03)		442 (25.18)		281 (20.07)				
	Middle or high school	2573 (30.55)		1843 (34.99)		529 (30.14)		201 (14.36)				
	College or above	208 (2.47)		177 (3.36)		21 (1.2)		10 (0.71)				
**Smoking status^b^, n (%)**												.04
	Nonsmoker	5064 (60.13)		3130 (59.43)		1079 (61.48)		855 (61.07)				
	Former smoker	762 (9.05)		454 (8.62)		172 (9.8)		136 (9.71)				
	Current smoker	2503 (29.72)		1623 (30.81)		486 (27.69)		394 (28.14)				
**Drinking status^b^, n (%)**												.002
	Nondrinker	5046 (59.91)		3148 (59.77)		1001 (57.04)		897 (64.07)				
	Former drinker	1172 (13.91)		740 (14.05)		261 (14.87)		171 (12.21)				
	Current drinker	2188 (25.98)		1368 (25.97)		491 (27.98)		329 (23.5)				
Depressive symptoms scores^b,c^, mean (SD)			8.27 (6.25)		7.67 (5.98)		8.45 (6.34)		10.41 (6.7)		<.001	
**Health conditions (yes), n (%)**												
	Diabetes	878 (10.42)		542 (10.29)		191 (10.88)		145 (10.36)		.78		
	Hypertension	3066 (36.4)		1866 (35.43)		598 (34.07)		602 (43)		<.001		
	Dyslipidemia	2459 (29.2)		1556 (29.54)		516 (29.4)		387 (27.64)		.37		
	Chronic kidney disease	480 (5.7)		294 (5.58)		110 (6.27)		76 (5.43)		.50		
**History of medication use (yes)^b^, n (%)**												
	Diabetes medications	267 (3.17)		184 (3.49)		54 (3.08)		29 (2.1)		.02		
	Hypertension medications	1385 (16.44)		906 (17.2)		241 (13.73)		238 (17.2)		.003		
	Lipid-lowering therapy	330 (3.92)		215 (4.08)		70 (3.99)		45 (3.3)		.33		
BMI (kg/m^2^), mean (SD)			23.21 (2.44)		23.19 (2.47)		23.32 (2.25)		23.13 (2.54)		.18	
**Blood pressure (mm Hg), mean (SD)**												
	Systolic pressure	131.11 (25.14)		130.36 (24.88)		129.57 (23.84)		135.65 (27.09)		<.001		
	Diastolic pressure	75.85 (12.13)		76.18 (12.08)		75.27 (12.02)		75.39 (12.43)		.02		
**Metabolic biomarkers^d^**												
	Fasting plasma glucose (mg/dL), mean (SD)	109.11 (33.48)		109.83 (36.14)		107.80 (29.48)		108.29 (28.25)		.16		
	HbA1c (%), mean (SD)	5.26 (0.78)		5.28 (0.83)		5.24 (0.70)		5.23 (0.67)		.24		
	Total cholesterol (mg/dL), mean (SD)	194.34 (37.71)		194.19 (37.93)		193.71 (36.69)		195.63 (38.23)		.50		
	Triglyceride (mg/dL), mean (SD)	127.75 (97.66)		131.32 (101.87)		125.14 (85.54)		118.71 (96.77)		.002		
	High-density lipoprotein (mg/dL), mean (SD)	51.78 (15.42)		50.93 (15.28)		52.25 (15.22)		54.11 (15.94)		<.001		
	Low-density lipoprotein (mg/dL), mean (SD)	117.92 (34.61)		117.91 (34.58)		117.22 (34.31)		118.90 (35.11)		.56		
	hs-CRP^e^ (mg/L), median (IQR)	1.00 (1.63)		1.04 (1.68)		0.92 (1.36)		1.01 (1.78)		.15		
	eGFR^f^ (mL/min/1.73 m^2^), mean (SD)	90.82 (34.11)		91.98 (36.09)		89.36 (31.72)		88.42 (28.99)		.02		

^a^*P* value was based on the chi-square test, ANOVA, or the Kruskal-Wallis test where appropriate.

^b^Missing data: 1 for the area of residence, 7 for educational level, 93 for smoking, 16 for drinking, 629 for depressive symptoms scores, 67 for diabetes medications, 37 for hypertension medications, and 178 for lipid-lowering therapy.

^c^Depressive symptom scores were measured using the 10-item Center for Epidemiology Scale for Depression, ranging from 0 to 30, with higher scores indicating a higher level of depressive symptom severity.

^d^Measured in the subpopulation of 5196 participants.

^e^hs-CRP: high-sensitivity C-reactive protein.

^f^eGFR: estimated glomerular ﬁltration rate.

### Associations of Social Isolation With Incident CVD

During the follow-up period between 2015 and 2019, a total of 746 incident CVDs occurred (heart disease: 450 cases and stroke: 336 cases). The incidence rate of CVD was 28.14 per 1000 person-years among individuals with consistently low social isolation, 34.81 per 1000 person-years among the fluctuating social isolation group, and 35.26 per 1000 person-years among those with consistently high social isolation. Table 2 shows that after adjusting for covariates in models 2 to 4, compared with individuals with consistently low social isolation (reference), those with fluctuating social isolation (adjusted HR 1.27, 95% CI 1.01-1.59; model 4) and those with consistently high social isolation (adjusted HR 1.45, 95% CI 1.13-1.85; model 4) had a higher risk for incident CVD. For CVD components, compared with the reference group, individuals with fluctuating social isolation (adjusted HR 1.59, 95% CI 1.16-2.17; model 4) and those with consistently high social isolation (adjusted HR 1.75, 95% CI 1.25-2.47; model 4) had a higher risk of stroke, but not heart disease (Table 2).

**Figure 2:**
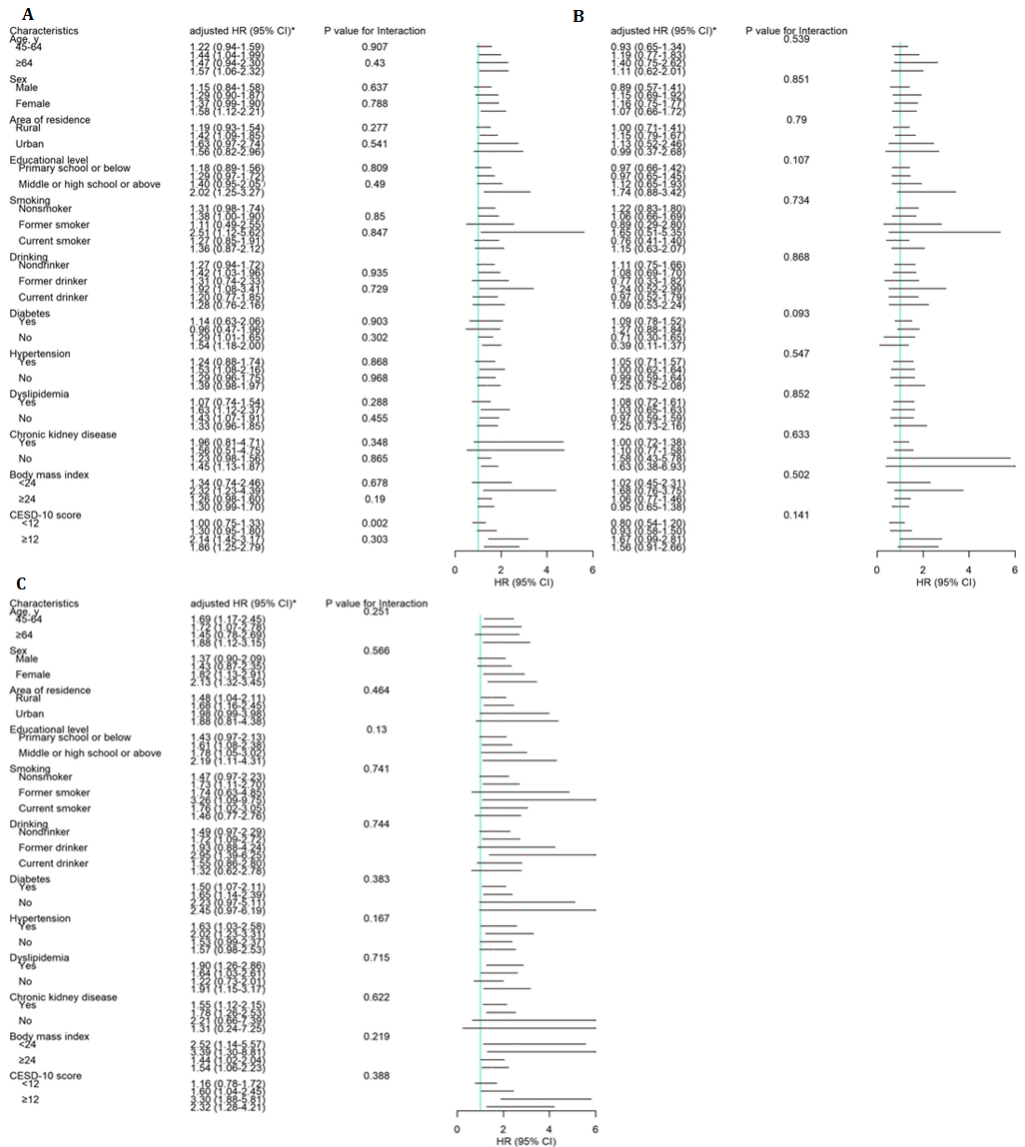
Longitudinal association of social isolation trajectories with incident cardiovascular disease (CVD) during the follow-up period. Graphs show hazard ratios (HRs) and 95% CIs for (A) CVD, (B) heart disease, and (C) stroke, adjusted for age; sex; residence; educational level; smoking status; drinking status; BMI; history of diabetes, hypertension, dyslipidemia, and chronic kidney disease; use of diabetes medications, hypertension medications, and lipid-lowering therapy; and depressive symptoms scores. *Consistently low social isolation (reference).

As shown in Figure 2, the association between the social isolation trajectory group and incident CVD events was not moderated by age, sex, residence, smoking status, drinking status, diabetes, hypertension, dyslipidemia, chronic kidney disease, BMI, and depressive symptoms scores. The association between consistently high social isolation and incident CVD was significant among participants with middle or high school or above educational level (adjusted HR 2.02, 95% CI 1.25-3.27; *P*=.03 for interaction). Moreover, we assessed the risk of incident CVD between fluctuating and consistently high social isolation subgroups. As shown in Table 3, we found that compared with individuals with fluctuating social isolation, those with consistently high social isolation were not significantly associated with an increased risk of incident CVD (*P*>.05).

Intercorrelations of the metabolic biomarkers used in this study are presented in Table S3 in Multimedia Appendix 1. Sensitivity analyses showed that after further adjusting for metabolic biomarkers, the associations of fluctuating social isolation and consistently high social isolation with incident stroke remained statistically significant (Table S4 in Multimedia Appendix 1). Our findings were not largely attenuated by health conditions and behaviors at wave 3 (Table S5 in Multimedia Appendix 1), and similar results were found when complete data analyses were performed (Table S6 in Multimedia Appendix 1) or using inverse-probability weighting (Table S7 in Multimedia Appendix 1).

**Table 2:** Cox proportional hazard ratios (HRs) for the association of social isolation trajectories with incident cardiovascular disease (CVD).

Outcome				Number of cases, n		Incidence rate (per 1000 person-years)		HR (95% CI)						
								Model 1^a^		Model 2^b^		Model 3^c^		Model 4^d^
**CVD**														
	**Social isolation trajectory group**													
		Consistently low	428		28.14		1.00 (reference)		1.00 (reference)		1.00 (reference)		1.00 (reference)	
		Fluctuating	175		34.81		1.26 (1.02-1.56)		1.26 (1.02-1.56)		1.25 (1.00-1.55)		1.27 (1.01-1.59)	
		Consistently high	143		35.26		1.45 (1.16-1.80)		1.40 (1.11-1.76)		1.38 (1.09-1.75)		1.45 (1.13-1.85)	
**Heart disease**														
	**Social isolation trajectory group**													
		Consistently low	273		17.77		1.00 (reference)		1.00 (reference)		1.00 (reference)		1.00 (reference)	
		Fluctuating	103		20.24		1.06 (0.83-1.47)		1.07 (0.77-1.49)		1.04 (0.74-1.43)		1.03 (0.75-1.40)	
		Consistently high	74		18.08		1.10 (0.83-1.47)		1.11 (0.83-1.48)		1.06 (0.79-1.43)		1.11 (0.78-1.58)	
**Stroke**														
	**Social isolation trajectory group**													
		Consistently low	181		11.60		1.00 (reference)		1.00 (reference)		1.00 (reference)		1.00 (reference)	
		Fluctuating	80		15.41		1.43 (1.06-1.94)		1.43 (1.05-1.93)		1.44 (1.06-1.96)		1.59 (1.16-2.17)	
		Consistently high	75		18.08		1.83 (1.36-2.47)		1.66 (1.21-2.27)		1.68 (1.21-2.32)		1.75 (1.25-2.47)	

^a^Model 1 was an unadjusted model.

^b^Model 2 was adjusted for age and sex.

^c^Model 3 was adjusted for age, sex, residence, educational level, smoking status, and drinking status.

^d^Model 4 was adjusted as model 3 plus BMI; history of diabetes, hypertension, dyslipidemia, chronic kidney disease; use of diabetes medications, hypertension medications, lipid-lowering therapy; and depressive symptoms scores.

**Table 3:** Cox proportional hazard ratios (HRs) for the association of social isolation trajectories with incident cardiovascular disease (CVD).

Outcome				Number of cases, n		Incidence rate (per 1000 person-years)		HR (95% CI)						
								Model 1^a^		Model 2^b^		Model 3^c^		Model 4^d^
**CVD**														
	**Social isolation trajectory group**													
		Fluctuating	175		34.81		1.00 (reference)		1.00 (reference)		1.00 (reference)		1.00 (reference)	
		Consistently high	143		35.26		1.15 (0.88-1.48)		1.10 (0.83-1.45)		1.11 (0.84-1.47)		1.12 (0.83-1.50)	
**Heart disease**														
	**Social isolation trajectory group**													
		Fluctuating	103		20.24		1.00 (reference)		1.00 (reference)		1.00 (reference)		1.00 (reference)	
		Consistently high	74		18.08		0.96 (0.66-1.40)		0.96 (0.64-1.43)		0.97 (0.65-1.46)		1.08 (0.70-1.65)	
**Stroke**														
	**Social isolation trajectory group**													
		Fluctuating	80		15.41		1.00 (reference)		1.00 (reference)		1.00 (reference)		1.00 (reference)	
		Consistently high	75		18.08		1.28 (0.90-1.81)		1.19 (0.82-1.73)		1.20 (0.82-1.75)		1.09 (0.74-1.62)	

^a^Model 1 was an unadjusted model.

^b^Model 2 was adjusted for age and sex.

^c^Model 3 was adjusted for age, sex, residence, educational level, smoking status, and drinking status.

^d^Model 4 was adjusted as model 3 plus BMI; history of diabetes, hypertension, dyslipidemia, chronic kidney disease; use of diabetes medications, hypertension medications, and lipid-lowering therapy; and depressive symptoms scores.

## Discussion

### Principal Findings

In this cohort study of middle-aged and older Chinese adults, individuals in the fluctuating and consistently high social isolation trajectory groups had a 27% and 45% higher risk for developing incident CVD than those with consistently low social isolation. The association between consistently high social isolation and incident CVD remained statistically significant after demographics, baseline health behaviors and conditions, and baseline depressive symptoms (including loneliness measurement) had been taken into account in multivariable models. However, after further adjusting for metabolic biomarkers, only the associations of fluctuating social isolation and consistently high social isolation with incident stroke remained statistically significant.

Several cross-sectional and longitudinal studies have suggested that the presence of social isolation is associated with a higher risk of coronary heart disease, heart failure [9], stroke [10], CVD diagnoses [38-40], and CVD risks [41]. Nagayoshi et al [10] demonstrated that community-dwelling men and women in the United States who reported having a small social network had an approximately 40% greater risk of incident stroke compared with their counterparts who reported a large social network, even after adjusting for demographics, socioeconomic variables, behavioral risk factors, and major stroke risk factors. Freak-Poli et al [40] reported a positive association between social isolation and incident CVDs, particularly stroke. However, these studies only assessed social isolation at a single time point, which may not provide a complete picture of the role of social isolation over the life span. In reality, the level of exposure to social isolation may be dynamic and vary over time [19]. Our study observed that 62.54% (5267/8422) of the study population had consistently low social isolation, 16.62% (1400/8422) had consistently high social isolation, and 20.83% (1755/8422) had fluctuating social isolation over the exposure period. Research on the longitudinal association between social isolation trajectories and CVD events is limited. Our study used repeated measures of social isolation and found that repeated occurrences of social isolation (ie, consistently high social isolation) may increase the risk of incident CVD, particularly stroke. The potential mechanisms underlying the association include elevated adrenaline levels in the blood, increased sympathetic nervous system activity, dysregulated heart rate and blood pressure, and modulated cardiovascular reactivity, as well as the activation of the hypothalamic-pituitary-adrenocortical axis and the sympathetic nervous system, or dysregulated inflammation [14,15,42]. In addition, social isolation is associated with unhealthy behavioral factors (eg, smoking, drinking, obesity, and lack of medication compliance) and psychological status (eg, depression), which may increase vascular risk and are considered CVD risk factors [43-47]. Consistent with previous literature [42,48-50], our study also observed that individuals in the consistently high social isolation group were more likely to be older, be female, live in more deprived areas, have higher systolic pressure, have higher depressive symptoms scores, and have poor medication compliance relative to those with consistently low social isolation. Our adjusted Cox regression models indicated that these factors might modestly explain the observed association between social isolation and CVD events. Furthermore, our sensitivity analysis, which was additionally adjusted for health conditions and behaviors at wave 3, resulted in attenuated risk estimates. Similarly, a large-scale prospective study using data from the UK Biobank reported that the association of social isolation with acute myocardial infarction and stroke was attenuated after adjusting for demographics, biological factors, health behaviors, and depressive symptoms [11]. Another large-scale prospective study using data from the UK Biobank and Million Women’s study found that after controlling for established cardiovascular risk factors (eg, age, sex, and self-rated health) and health behaviors, no significant overall association between social isolation and nonfatal coronary heart disease and stroke was observed. However, in contrast, social isolation had clear associations with fatal coronary heart disease and stroke [49]. Furthermore, the association between social isolation and increased levels of inflammatory biomarkers, such as hs-CRP, are well documented [51,52]. In this study, after adjusting for hs-CRP and other metabolic biomarkers, the association of fluctuating social isolation and consistently high social isolation with incident stroke remained significant, suggesting that these findings are robust. Apart from the abovementioned mechanisms linking social isolation and CVD events, there may also be trajectory-specific mechanisms that require further investigation.

In addition, our study found that the association between the social isolation trajectory group and incident CVD events was not moderated by age, sex, residence, smoking status, drinking status, diabetes, hypertension, dyslipidemia, chronic kidney disease, BMI, and depressive symptoms scores. Only educational level modestly moderated the association between social isolation and incident CVD, with the highest HR observed in individuals with middle or high school education or above educational level. Similarly, Cené et al [9] did not observe the moderation effects of age, race, and ethnicity on the association between social isolation and incident heart failure among older women in the United States. Bu et al [39] reported no evidence of a moderation effect of age, sex, socioeconomic status, and CVD risk (established by obesity, high cholesterol, hypertension, diabetes, smoking, diet, physical activity, abnormal sleep, and depression) on the association between social isolation and CVD events among participants from the ELSA. Although we have no clear explanation for this finding, our study indicates that the social isolation trajectory and incident CVD may not differ in individuals with different ages, sexes, residences, BMI, or other abovementioned characteristics. However, for individuals with higher educational levels, the influences of social isolation trajectory on incident CVD may be greater than that for those with lower education levels. Nevertheless, the observed moderation effects of educational level or other unobserved findings still require further investigation.

Our study also found that individuals in the consistently high social isolation group did not have a higher risk of incident CVD than those with fluctuating social isolation. This result may be because individuals with fluctuating social isolation may also have increased risks of other health conditions, such as depressive symptoms, hypertension, and obesity, which may, in turn, increase their risk of developing CVDs [53,54]. Moreover, the fluctuating social isolation trajectory group in our study included a subgroup of individuals who were older, lived in rural areas, and had higher depressive symptoms scores than those with consistently low social isolation. On the basis of our findings, we recommend early identification and lifestyle interventions for socially isolated individuals as a means of combating CVDs and promoting healthy aging, with particular attention paid to the persistent occurrence of social isolation. Future research is needed to examine how frequently social isolation should be assessed and how many assessments would be sufficient to present the course of social isolation to determine high-risk groups for CVDs.

### Strengths and Limitations

The strengths of our study include its prospective design, repeated measures of social isolation, a long follow-up period, and a wide range of covariates that include health conditions and behaviors and metabolic biomarkers. Repeated social isolation measurements over a long exposure period can minimize the likelihood of reverse causation or the potential bidirectionality of the estimated association between social isolation and CVD event. However, this study has several limitations. First, the diagnosis of CVD relied on self-reported data, which may be subject to reporting bias. Although medical records were not available in the CHARLS [33], previous large-scale studies using data from the Health and Retirement Study [55] or the ELSA [56] found that self-reported CVDs correspond well with CVDs coded according to the International Classification of Diseases in medical records. Second, individuals with missing social isolation measures and those who experienced CVD during the exposure assessment period were excluded from the analyses, which may limit the generalizability of our findings to middle-aged and older adults. Third, the predefined trajectory group definition of social isolation in our study may be only one possible representation of the accumulative progression of social isolation over time. Fourth, our study sample only included Chinese adults, and therefore our study findings may not be fully generalizable to other ethnicities. Fifth, although our social isolation index was based on previous research [22,23], it was not a standardized scale or instrument. Sixth, our analyses did not consider physical activity as a covariate because it was not assessed using a standardized scale and may be an intermediate variable between social isolation and CVD [57-61]. Finally, in one of our sensitivity analyses presented in Table S4 in Multimedia Appendix 1, all metabolic biomarkers were adjusted to test the robustness of our main findings. However, some of these markers appeared to be moderately correlated, which may have led to collinearity issues.

### Conclusions

In this cohort study, both fluctuating and consistently high social isolation exposure were associated with a higher risk of incident CVD among middle-aged and older Chinese adults, especially for stroke. Our findings provide new evidence supporting the longitudinal link between social isolation trajectories and CVD, highlighting the importance of routine screening for social isolation, and providing preventive or intervention strategies to both fluctuate isolated or consistently isolated individuals to reduce the incidence of CVD and promote healthy aging. In addition, our results suggest that efforts to improve social connectedness and reduce social isolation among middle-aged and older adults are recommended.

## Appendix

Multimedia Appendix 1 Details of the measured variables, trajectory definitions, baseline survey characteristics, intercorrelations of metabolic markers, and sensitivity analyses.

## Abbreviations

CESD-10: 10-item Center for Epidemiology Scale for Depression
CHARLS: China Health and Retirement Longitudinal Study
CVD: cardiovascular disease
ELSA: English Longitudinal Study of Ageing
HR: hazard ratio
hs-CRP: high-sensitivity C-reactive protein
PPS: probability-proportional-to-size

## Data Availability

The original data sets are publicly available on the website of the China Health and Retirement Longitudinal Study (CHARLS) [62]. The data sets generated and analyzed during this study are available from the corresponding author upon reasonable request.
