# Supplementary material for: Evaluation of Social Isolation Trajectories and Incident Cardiovascular Disease Among Middle-Aged and Older Adults in China: National Cohort Study
Source: JMIR Public Health Surveill. 2023 Jun 30;9:e45677. doi: 10.2196/45677 (PMC10365588; doi:10.2196/45677)
Supplement: Multimedia Appendix 1 [file publichealth_v9i1e45677_app1.docx]

Multimedia Appendix 1.

Title: Evaluation of social isolation trajectories and incident cardiovascular disease among middle-aged and older adults in China: a national cohort study

Methods. Details of the measured variables in this study.

Table S1**.** Trajectory definitions for the main results.

Table S2**.** Characteristics of included and excluded individuals at baseline survey.

**Table S3.** Intercorrelations (r) of the metabolic biomarkers used in this study.

Table S4**.** Sensitivity analysis results for the association of social isolation trajectories with incident CVD in subpopulations of 5196 participants with metabolic biomarkers measurements.

Table S5**.** Sensitivity analysis results for the association of social isolation trajectories with incident CVD after additionally adjusting for health conditions and behaviors at wave 3.

Table S6**.** Sensitivity analysis results for the association of social isolation trajectories with incident CVD in subpopulations of 7472 participants with complete data.

Table S7**.** Sensitivity analysis results for the association of social isolation trajectories with incident CVD using inverse-probability weighting.

eMethods. Details of the measured variables in this study

*Definitions of social isolation*

The index of social isolation includes 4 indicators: living arrangement (1 point for living alone), marital status (1 point for currently unmarried, including separated, divorced, widowed, and never married), contacts with children (1 point for contacting with children in person or by phone/email less than once a week), and social participation (1 point for participating in any social activities less than once a month) [22,23]. Six types of social activities during the last month from the CHARLS database were extracted, including (1) interacting with friends; (2) playing Mahjong, chess, or cards, or going to a community club; (3) going to a sport, social, or other kinds of club; (4) taking part in a community-related organization; (5) attending an educational or training course; and (6) doing voluntary or charity work. A total score of social isolation ranged from 0 to 4 by summing these dichotomized indicators, with higher scores representing a greater level of social isolation.

*Definitions of hypertension, dyslipidemia, diabetes, and chronic kidney disease*

Hypertension was defined as self-reported history of hypertension, current use of the antihypertensive medication, systolic blood pressure ≥140 mmHg, or diastolic blood pressure ≥90 mmHg according to the Chinese hypertension guidelines [24]. Dyslipidemia was defined as self-reported history of dyslipidemia, current use of the lipid-lowering medication, total cholesterol 240 mg/dL, low-density lipoprotein cholesterol ≥160 mg/dL, high-density lipoprotein cholesterol <40 mg/dL, or triglycerides ≥150 mg/dL according to the Chinese guidelines for the management of dyslipidemia in adults [25]. Diabetes was defined as self-reported history of diabetes, current use of anti-diabetic medication, a fasting plasma glucose level ≥126 mg/dL or an HbA1c level ≥6.5% according to the 2010 American Diabetes Association (ADA) guidelines [26]. Chronic kidney disease was deﬁned as self-reported history of physician-diagnosed chronic kidney disease and/or estimated glomerular ﬁltration rate (eGFR ) < 60 mL/min/1.73 m^2^ [27].

*Body mass index*

Body mass index (BMI) was calculated as the weight in kilograms divided by the square of height in meters (kg/m^2^).

*Use of medications or therapies for hypertension, diabetes, and dyslipidemia*

Are you now taking any of the following treatments to treat hypertension/diabetes/dyslipidemia or its complications? Responses were categorized into 1=yes and 2=no.

| Table S1**.** Trajectory definitions for the main results | | |
| --- | --- | --- |
| Consistently high | | |
| Wave 1 | Wave 2 | Wave3 |
| + | + | + |
| Consistently low | | |
| Wave 1 | Wave 2 | Wave3 |
| - | - | - |
| Fluctuating | | |
| Wave 1 | Wave 2 | Wave 3 |
| + | + | - |
| + | - | - |
| + | - | + |
| - | + | + |
| - | - | + |
| - | + | - |

| Table S2**.**Characteristics of included and excluded individuals at baseline survey. | | |
| --- | --- | --- |
| **Characteristics** | **Included (N=8422)** | **Excluded^a^ (N=9286)** |
| Age, mean (SD), y | 59.76 (10.33) | 60.53 (10.17) |
| Sex |  |  |
| Male | 4219 (50.1) | 4524 (48.8) |
| Female | 4203 (49.9) | 4759 (51.2) |
| Missing data | 0 | 3 |
| Area of residence |  |  |
| Rural | 6617 (78.6) | 5302 (89.9) |
| Urban | 1803 (21.4) | 594 (10.1) |
| Missing data | 1 | 3390 |
| Educational level, n (%) |  |  |
| No formal education | 3698 (43.9) | 2188 (47.9) |
| Primary school or below | 1936 (23.0) | 1048 (23.0) |
| Middle or high school | 2573 (30.6) | 1079 (23.6) |
| College or above | 208 (2.5) | 249 (5.5) |
| Missing data | 7 | 4722 |
| Smoking status, n (%) |  |  |
| Nonsmoker | 5064 (60.8) | 5571 (60.3) |
| Former smoker | 762 (9.1) | 829 (9.0) |
| Current smoker | 2503 (30.1) | 2830 (30.7) |
| Missing data | 93 | 56 |
| Drinking status, n (%) |  |  |
| Nondrinker | 5046 (60.0) | 5536 (60.5) |
| Former drinker | 1172 (13.9) | 1326 (14.5) |
| Current drinker | 2188 (26.0) | 2284 (25.0) |
| Missing data | 16 | 140 |
| Depressive symptoms scores, mean (SD) | 8.27 (6.25) | 8.64 (6.43) |
| BMI, kg/m^2^, mean (SD) | 23.21 (2.44) | 23.00 (2.88) |

^a^ Excluded individuals were those lost to follow-up or not meeting the inclusion and exclusion criteria.

| Table S3**.** Intercorrelations (r) of the metabolic biomarkers used in this study. | | | | | | | | |
| --- | --- | --- | --- | --- | --- | --- | --- | --- |
| **Variables** | **Pearson Correlation Coefficient (r)** | | | | | | | |
|  | **1** | **2** | **3** | **4** | **5** | **6** | **7** | **8** |
| Fasting plasma glucose | 1 |  |  |  |  |  |  |  |
| HbA1c | 0.66^**^ | 1 |  |  |  |  |  |  |
| Total Cholesterol | 0.10^**^ | 0.13^**^ | 1 |  |  |  |  |  |
| Triglyceride | 0.26^**^ | 0.11^**^ | 0.27^**^ | 1 |  |  |  |  |
| High-density lipoprotein | -0.12^**^ | -0.05^**^ | 0.21^**^ | -0.43^**^ | 1 |  |  |  |
| Low-density lipoprotein | 0.01 | 0.09^**^ | 0.83^**^ | -0.12^**^ | 0.08^**^ | 1 |  |  |
| hs-CRP | 0.07^**^ | 0.05^**^ | -0.03^**^ | -0.02 | -0.05^**^ | -0.002 | 1 |  |
| eGFR | 0.03 | -0.02 | 0.01 | 0.02 | -0.05^**^ | 0.02 | 0.03^**^ | 1 |

^**^ *P* <0.05.

| Table S4**.** Sensitivity analysis results for the association of social isolation trajectories with incident CVD in subpopulations of 5196 participants with metabolic biomarkers measurements. | | | |
| --- | --- | --- | --- |
| **Social isolation trajectory group** | **HR (95% CI)** | | |
|  | **Cardiovascular disease** | **Heart disease** | **Stroke** |
| Consistently low | 1.00 (reference) | 1.00 (reference) | 1.00 (reference) |
| Fluctuating |  |  |  |
| Model 4 | 1.27 (1.01-1.59) | 1.03 (0.75-1.40) | 1.59 (1.16-2.17) |
| Model adjusted as Model 4 plus |  |  |  |
| Fasting plasma glucose | 1.25 (0.96-1.63) | 0.92 (0.64-1.32) | 1.72 (1.19-2.49) |
| HbA1c | 1.26 (0.97-1.63) | 0.96 (0.67-1.37) | 1.66 (1.15-2.41) |
| Total Cholesterol | 1.24 (0.95-1.61) | 0.92 (0.64-1.33) | 1.69 (1.17-2.44) |
| Triglyceride | 1.24 (0.95-1.61) | 0.92 (0.64-1.32) | 1.69 (1.17-2.45) |
| High-density lipoprotein | 1.25 (0.96-1.63) | 0.93 (0.65-1.33) | 1.72 (1.19-2.49) |
| Low-density lipoprotein | 1.25 (0.96-1.62) | 0.92 (0.64-1.33) | 1.71 (1.18-2.47) |
| hs-CRP | 1.25 (0.96-1.63) | 0.93 (0.65-1.330 | 1.71 (1.18-2.48) |
| eGFR | 1.25 (0.96-1.62) | 0.92 (0.64-1.33) | 1.71 (1.18-2.47) |
| All biomarkers | 1.25 (0.96-1.62) | 0.92 (0.64-1.33) | 1.72 (1.18-2.49) |
| Consistently high |  |  |  |
| Model 4 | 1.45 (1.13-1.85) | 1.11 (0.78-1.58) | 1.75 (1.25-2.47) |
| Model adjusted as Model 4 plus |  |  |  |
| Fasting plasma glucose | 1.32 (0.99-1.79) | 0.82 (0.52-1.27) | 1.98 (1.32-2.96) |
| HbA1c | 1.31 (0.98-1.77) | 0.83 (0.53-1.28) | 1.89 (1.27-2.83) |
| Total Cholesterol | 1.31 (0.97-1.76) | 0.81 (0.52-1.26) | 1.92 (1.29-2.87) |
| Triglyceride | 1.31 (0.97-1.76) | 0.81 (0.52-1.26) | 1.93 (1.29-2.88) |
| High-density lipoprotein | 1.33 (0.98-1.78) | 0.82 (0.53-1.27) | 1.96 (1.31-2.94) |
| Low-density lipoprotein | 1.32 (0.98-1.78) | 0.82 (0.53-1.27) | 1.95 (1.31-2.92) |
| hs-CRP | 1.32 (0.98-1.78) | 0.82 (0.53-1.27) | 1.95 (1.31-2.92) |
| eGFR | 1.32 (0.98-1.78) | 0.82 (0.53-1.27) | 1.96 (1.31-2.94) |
| All biomarkers | 1.34 (1.00-1.80) | 0.81 (0.52-1.26) | 2.02 (1.35-3.03) |

Abbreviation: HR, hazard ratio; CI, confidence interval; hs-CRP, high-sensitivity C-reactive protein; eGFR, estimated glomerular ﬁltration rate; CVD, cardiovascular disease.

Model 4 was adjusted for age, sex, residence, educational level, smoking status, drinking status, body mass index, history of diabetes, hypertension, dyslipidemia, chronic kidney disease; use of diabetes medications, hypertension medications, lipid-lowering therapy; and depressive symptoms scores.

| Table S5**.** Sensitivity analysis results for the association of social isolation trajectories with incident CVD after additionally adjusting for health conditions and behaviors at wave 3. | | | |
| --- | --- | --- | --- |
| **Social isolation trajectory group** | **HR (95% CI)** | | |
|  | **Cardiovascular disease** | **Heart disease** | **Stroke** |
| Consistently low | 1.00 (reference) | 1.00 (reference) | 1.00 (reference) |
| Fluctuating |  |  |  |
| Model 4 | 1.27 (1.01-1.59) | 1.03 (0.75-1.40) | 1.59 (1.16-2.17) |
| Model adjusted as Model 4 plus |  |  |  |
| Health conditions and behaviors at T3 | 1.21 (0.96-1.52) | 1.00 (0.73-1.38) | 1.47 (1.06-2.03) |
| Consistently high |  |  |  |
| Model 4 | 1.45 (1.13-1.85) | 1.11 (0.78-1.58) | 1.75 (1.25-2.47) |
| Model adjusted as Model 4 plus |  |  |  |
| Health conditions and behaviors at T3 | 1.35 (1.05-1.74) | 1.07 (0.74-1.53) | 1.59 (1.12-2.28) |

Abbreviation: HR, hazard ratio; CI, confidence interval; CVD, cardiovascular disease.

Model 4 was adjusted for age, sex, residence, educational level, smoking status, drinking status, body mass index, history of diabetes, hypertension, dyslipidemia, chronic kidney disease; use of diabetes medications, hypertension medications, lipid-lowering therapy; and depressive symptoms scores.

| Table S6**.** Sensitivity analysis results for the association of social isolation trajectories with incident CVD in subpopulations of 7472 participants with complete data. | | | | | | |
| --- | --- | --- | --- | --- | --- | --- |
| **Outcome** | **No. of cases** | **Incidence Rate, per 1000 person-years** | **HR (95% CI)** | | | |
|  |  |  | **Model 1** | **Model 2** | **Model 3** | **Model 4** |
| Cardiovascular disease |  |  |  |  |  |  |
| Social isolation trajectory group |  |  |  |  |  |  |
| Consistently low | 394 | 28.17 | 1.00 (reference) | 1.00 (reference) | 1.00 (reference) | 1.00 (reference) |
| Fluctuating | 161 | 34.56 | 1.26 (1.01-1.58) | 1.26 (1.01-1.57) | 1.27 (1.01-1.59) | 1.27 (1.01-1.59) |
| Consistently high | 127 | 35.55 | 1.51 (1.20-1.90) | 1.43 (1.13-1.82) | 1.45 (1.13-1.85) | 1.45 (1.13-1.85) |
| Heart disease |  |  |  |  |  |  |
| Social isolation trajectory group |  |  |  |  |  |  |
| Consistently low | 254 | 17.98 | 1.00 (reference) | 1.00 (reference) | 1.00 (reference) | 1.00 (reference) |
| Fluctuating | 92 | 19.50 | 1.05 (0.77-1.42) | 1.05 (0.77-1.43) | 1.04 (0.77-1.42) | 1.03 (0.75-1.40) |
| Consistently high | 67 | 18.61 | 1.14 (0.82-1.58) | 1.14 (0.81-1.60) | 1.13 (0.80-1.60) | 1.11 (0.78-1.58) |
| Stroke |  |  |  |  |  |  |
| Social isolation trajectory group |  |  |  |  |  |  |
| Consistently low | 164 | 11.42 | 1.00 (reference) | 1.00 (reference) | 1.00 (reference) | 1.00 (reference) |
| Fluctuating | 77 | 16.01 | 1.53 (1.12-2.09) | 1.51 (1.11-2.07) | 1.54 (1.13-2.11) | 1.59 (1.16-2.17) |
| Consistently high | 64 | 17.51 | 1.89 (1.38-2.60) | 1.68 (1.20-2.34) | 1.73 (1.23-2.43) | 1.75 (1.25-2.47) |

Abbreviation: HR, hazard ratio; CI, confidence interval; CVD, cardiovascular disease.

Model 1 was an unadjusted model.

Model 2 was adjusted for age and sex.

Model 3 was adjusted for age, sex, residence, educational level, smoking status, and drinking status.

Model 4 was adjusted as Model 3 plus body mass index; history of diabetes, hypertension, dyslipidemia, chronic kidney disease; use diabetes medications, hypertension medications, lipid-lowering therapy; and depressive symptoms scores.

| Table S7. Sensitivity analysis results for the association of social isolation trajectories with incident CVD using inverse-probability weighting. | | | | |
| --- | --- | --- | --- | --- |
| **Outcome** | **HR (95% CI)** | | | |
|  | **Model 1** | **Model 2** | **Model 3** | **Model 4** |
| Cardiovascular disease |  |  |  |  |
| Social isolation trajectory group |  |  |  |  |
| Consistently low | 1.00 (reference) | 1.00 (reference) | 1.00 (reference) | 1.00 (reference) |
| Fluctuating | 1.29 (0.99-1.67) | 1.22 (0.93-1.60) | 1.20 (0.91-1.58) | 1.31(1.01-1.68) |
| Consistently high | 1.36 (1.07-1.74) | 1.36 (1.07-1.74) | 1.32 (1.03-1.69) | 1.22 (0.91-1.64) |
| Heart disease |  |  |  |  |
| Social isolation trajectory group |  |  |  |  |
| Consistently low | 1.00 (reference) | 1.00 (reference) | 1.00 (reference) | 1.00 (reference) |
| Fluctuating | 1.13 (0.80-1.60) | 1.11 (0.77-1.59) | 1.12 (0.78-1.62) | 1.15 (0.79-1.67) |
| Consistently high | 1.31 (0.96-1.78) | 1.32 (0.97-1.79) | 1.31 (0.95-1.79) | 1.19 (0.86-1.64) |
| Stroke |  |  |  |  |
| Social isolation trajectory group |  |  |  |  |
| Consistently low | 1.00 (reference) | 1.00 (reference) | 1.00 (reference) | 1.00 (reference) |
| Fluctuating | 1.34 (1.02-1.76) | 1.33 (1.01-1.75) | 1.33 (1.01-1.76) | 1.41 (1.03-1.94) |
| Consistently high | 1.83 (1.36-2.47) | 1.39 (1.04-1.86) | 1.39 (1.03-1.87) | 1.46 (1.10-1.95) |

Abbreviation: HR, hazard ratio; CI, confidence interval; CVD, cardiovascular disease.

Model 1 was an unadjusted model.

Model 2 was adjusted for age and sex.

Model 3 was adjusted for age, sex, residence, educational level, smoking status, and drinking status.

Model 4 was adjusted as Model 3 plus body mass index; history of diabetes, hypertension, dyslipidemia, chronic kidney disease; use diabetes medications, hypertension medications, lipid-lowering therapy; and depressive symptoms scores.
